# Together we stand, apart we fall: how cell-to-cell contact/interplay provides resistance to ferroptosis

**DOI:** 10.1038/s41419-020-02994-w

**Published:** 2020-09-23

**Authors:** Milica Vucetic, Boutaina Daher, Shamir Cassim, Willian Meira, Jacques Pouyssegur

**Affiliations:** 1 Medical Biology Department, Centre Scientifique de Monaco (CSM), Monaco, Monaco; 2 University Côte d’Azur, Institute for Research on Cancer & Aging (IRCAN), CNRS, INSERM, Centre A. Lacassagne, Nice, France

**Keywords:** Cancer therapeutic resistance, Cell death

## Abstract

Contextualisation of the new type of cell death called “ferroptosis” opened a completely new avenue for the development of anti-cancer therapies. Cumulative fundamental research dating back to the mid-20th century, crowned by the extraordinary work of the group led by Dr. Stockwell from Columbia University in 2012, finally got its candidature to be applied in the clinical settings. Although the potential for clinical importance is undoubtedly growing every day, as showed by the increasing number of papers dealing with ferroptosis and its applications, long experience of cancer research and treatment taught us that caution is still necessary. The plasticity of the tumour cells, particularly acute, along with its involvement in the resistance mechanisms, that have been seen, to greater or lesser extent, for almost all currently used therapies, represents the biggest fascinations in biomedical research field and also the biggest challenge to achieving cures in cancer patients. Accordingly, the main features of fundamental research have to be vigilance and anticipation. In this review, we tried to summarize the literature data, accumulated in the past couple of years, which point out the pitfalls in which “ferroptosis inducers” can fall if used prematurely in the clinical settings, but at the same time can provide a great advantage in the exhausting battle with cancer resistance. This is the first comprehensive review focusing on the effects of the cell-to-cell contact/interplay in the development of resistance to ferroptosis, while the contribution of cell-born factors has been summarized previously so here we just listed them.

## Facts

- Newly contextualized type of cell death termed “ferroptosis”, although highly promising as a strategy for eradication of the tumour cells, still has not been fully understood in the in vivo conditions.
- Cell-to-cell contact influences the sensitivity of the cancer cells to ferroptosis
- Cancer cell resistance to ferroptosis at higher density has been ascribed to the activation of the Hippo pathway and its intersections with other signalling pathways/ processes such as mTORC1, EMT, etc.
- The Hippo pathway has been described to contribute to cell density effects, although this seemingly just applies to adherent cancer cells, and as such might be cell context dependent.
- Besides direct contact, metabolic shuttling (cell-to-cell interplay) between the tumour and stromal cells fundamentally changes the response of these cells to ferroptosis induction.

## Open questions

- Is Hippo pathway the only/main mechanism involved in the high-density-induced resistance to ferroptosis of the adherent cells?
- Does the cell-to-cell contact of the non-adherent cells have the same effect on the ferroptosis sensitivity?
- How the cell-to-cell interplay can be used to enhance the efficacy of the ferroptosis inducers, other chemotherapeutics and/or immunotherapy?

## Introduction

The (patho)physiology of the cancer development and progression represents an extremely complex field, which prevents us, until this date, to form comprehensive definition of the disease. Surprisingly, in the review from 2002, Green and Evan^1^ hypothesized that the main driving force for all cancer types is “unique, very rare, simultaneous acquisition of the two cooperating conditions—deregulated cell proliferation and suppressed apoptosis”. According to the authors, all the other driving forces, seen on this “established cancer platform”, which consequently lead to divergences from the common evolutionary trajectory, are the result of the changing selective pressure in the expanding cell population. Although the difference between this two-hallmark and, widely accepted, six(ten)-hallmark^2,3^ description of cancer cell can be a matter of debate, when it comes to anti-cancer treatment, only one question is really important—how to specifically kill cancer cells?

Early studies dealing with the involvement of apoptosis in the neoplastic transformation and cancer progression^4^, together with cloning and characterization of the first oncogenes (such as *bcl-2*) and tumour suppressor genes (such as *p53*)^5^, sparked optimism in the cancer research community. Thus, the focus was placed on the understanding and exploiting strategies for (re)induction of the cell death program in cancers, and eventually, the development of new and more effective anti-cancer regiments^6–9^. In parallel, scientists were learning more about the complexity of the apoptosis, different intrinsic/extrinsic signals that can trigger this type of cell death, and unfortunately, the resistance mechanisms through the incredible power of the Darwinian selection.

Similar optimism, when it comes to anti-cancer therapeutics, appeared in the scientific community once again quite recently, when the group of Dr. Brent Stockwell at Columbia University contextualized a new type of cell death, called “ferroptosis”^10^. According to a growing number of data, initial optimism seems to be justified since ferroptosis-induction proved to be a highly promising strategy for eradication of the cancer cells. However, in order to avoid the trap of oversimplification, special attention has to be placed on the multiple potential mechanisms that confer resistance to this type of cell death.

The aim of this review is to summarize the recent findings suggesting that cell-to-cell contact and interplay impact tumour cell sensitivity to ferroptosis. Furthermore, we tried to place these effects in the context of cellular features that might be perceived as (only) cell-born treats, and to point out the most important avenues that remain to be examined.

## Ferroptosis—in the perspective

Although the contextualization of ferroptosis, as a new type of cell death happened just a couple years ago^10^, the research leading to this extraordinary discovery dates back several decades in the past and is marked by a couple of major milestones that paved the way to the ferroptosis concept as we know it today^11^. The first major discovery happened in the mid-20th century by Dr. Harry Eagle who, by investigating the nutritional requirements for survival and growth of mammalian cells in in vitro conditions, showed that the classified non-essential amino acid—cysteine (CySH), is not only essential for proliferation of cells in culture, but its absence inevitably leads to very specific type of cell death^12–14^. Two decades later, exceptional work of the Japanese group led by Dr Shiro Bannai, depicted the essential connection between CySH-starvation, depletion of intracellular glutathione (GSH) and consequent accumulation of reactive oxygen species (ROS), and ultimately, cell death^15^. Moreover, this study added up to the general knowledge of lipid-based oxidative damage of the cell that had been started independently by the groups of Dr. Peterson and Dr. Rothberg^16,17^ and continued until 80 s, when the process of lipid peroxidation was officially recognized as a major form of oxidative damage of lipid compartments within the cell, correlating with many different pathologies^18–20^. Last milestone in the ferroptosis research, which happened about the same time, is the seminal discovery, isolation and purification of *phospholipid hydroperoxide glutathione peroxidase 4* (PHGPX4 aka GPx4) by Dr. Fulvio Ursini and coworkers^21^. Importantly, the loss/inhibition of this enzyme leading to specific type of non-apoptotic cell death was actually the first step toward ferroptosis contextualization^22,23^. These four major milestones that happened over the 30-year long period had massive impact on our understanding of oxidative damage and its involvement in the process of cell death; however it took another 30 years until we were able to put the major parts of ferroptosis jigsaw together. For this we can be grateful to the work of the Dr. Stockwell’s and Dr Conrad’s groups, done in the 10-year long period from 2001 to 2012.

What we have learned about ferroptosis during the decade that followed? Ferroptosis is classified under “regulated” types of cell death relying on dedicated molecular machinery, and as such, can be induced/prevented by different pharmacological/genetic manipulations. On the other side, it is still not clear whether ferroptosis can be classified in the group of “programmed” cell deaths, considering that in contrast to the ‘clean’-apoptosis cell death, ferroptosis leads to a sort of explosive necrotic cell death able to induce an inflammatory response.

**Fig. 1:**
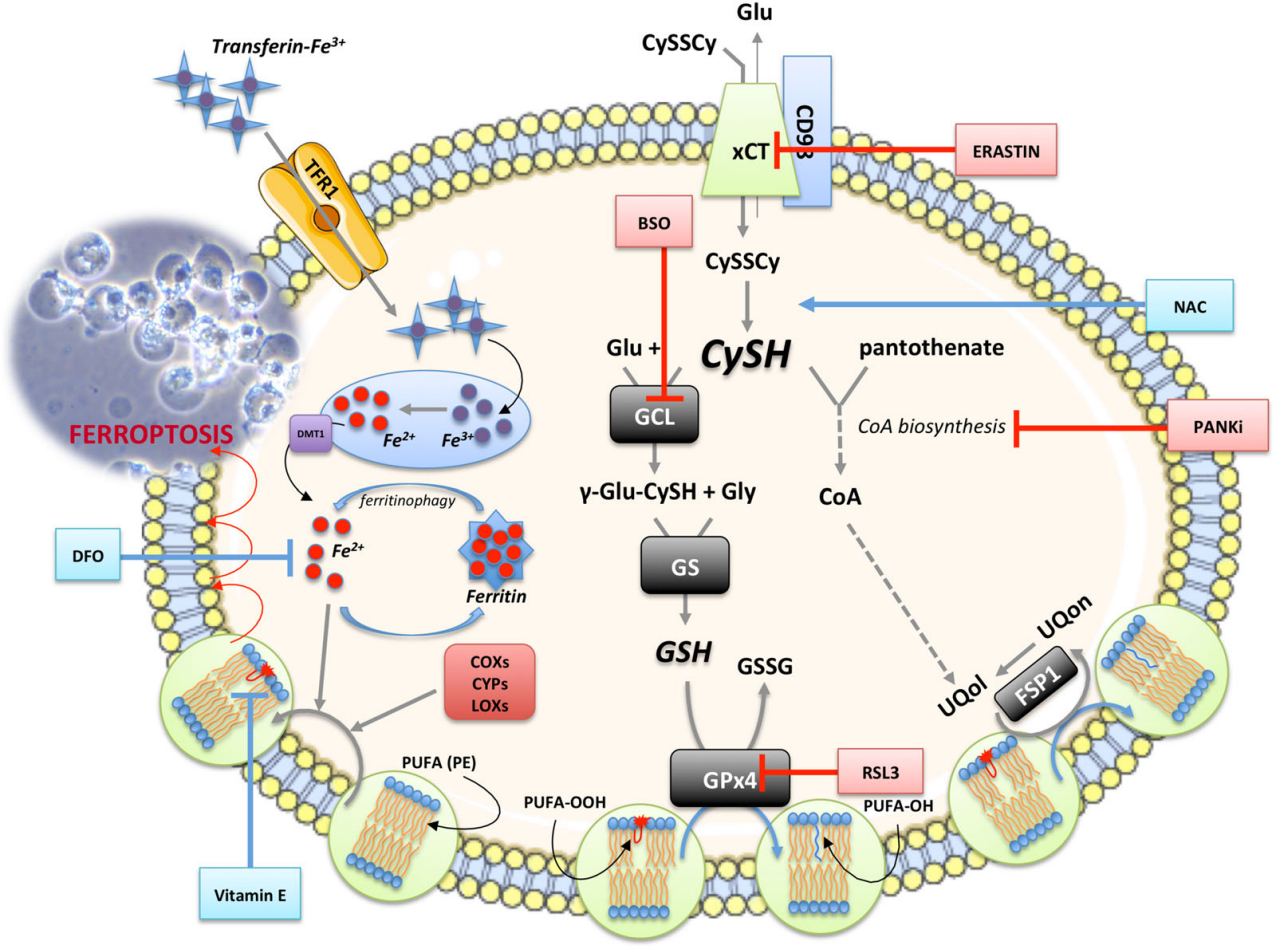
Ferroptosis overview. Under basal conditions, cancer cells take up cystine (CySSCy) via xCT transporter, reduce it and use it for many different purposes. One of the main roles of CySH in the cell is synthesis of glutathione (GSH). GSH serves as co-substrate for many antioxidant enzymes, including glutathione peroxidase 4 (GPx4). In the context of ferroptosis, the GPx4 plays an important role as neutralizer of oxidative damage in the membrane compartments of the cell. In the presence of ‘labile‘ Fe^2+^ ions (Fenton reaction), oxidants attack membrane polyunsaturated fatty acids (PUFA), such as phosphatidyl-ethanolamine (PE), converting them to highly toxic lipid peroxides (PUFA-OOH). Due to its high redox potency, the level of iron in the cell is kept under tight regulation. Extracellular Fe^3+^, bound to transport protein transferrin, is taken up by cells via transferrin receptor 1 (TFR1), transported into endosome where it undergoes reduction to Fe^2+^ by metalloreductases. Divalent metal transporter 1 (DMT1) mediates the transport of Fe^2+^ from the endosome into the cytoplasmic labile iron pool, where most of it is ligated by heme, bound in FeS clusters, or stored in the iron storage protein ferritin. However, small amount of free and catalytically active Fe^2+^ ions is present in the cytoplasm coming either directly from endosomes or from autophagic degradation of ferritin in the process known as ferritinophagy. In addition to being formed through non-specific propagation of radicals, oxidized lipids can also be synthesized in an enzymatically-regulated manner by cyclooxygenases (COXs), cytochrome p450s (CYPs), and lipoxygenases (LOXs). Under basal conditions, the level of PUFA-OOH is controlled by the action of GPx4 enzyme. This Se-peroxidase uses reducing power of GSH to convert toxic PUFA-OOH into non-toxic alcohol form (PUFA-OH), protecting membrane and cellular integrity. Alternatively, PUFA-OOH can be reduced by the ubiquinol (UQol). Thereby produced ubiquinon (UQon) is reduced back to UQol form by the action of ferroptosis suppressor protein 1 (FSP1). Blocking CySSCy import via xCT (erastin), synthesis of GSH (buthionine sulfoximine, BSO), GPx4 activity (RAS-selective lethal 3, RSL3) or the biosynthesis of UQol (PANKi), antioxidant balance within the cell is disturbed and accumulation of lipid peroxide leads to the lost of plasma membrane assembly, composition, structure, and finally cell osmotic necrotic collapse and death (ferroptosis). This type of the cell death is classified under ‘regulated‘ forms of cell death as some compounds can specifically prevent it. As an example, Vitamin E (tocopherol α) a lipophilic antioxidant that can incorporate to the membrane compartment and stop chain-formation of PUFA-OOH, N-acetylcysteine (NAC) can serve as an alternative source of cysteine, while deferoxamine (DFO) chelate ‘labile‘ metal ions including Fe^2+^. This figure was created using Servier Medical Art templates, which are licensed under a Creative Commons Attribution 3.0 Unported License; https://smart.servier.com.

The molecular machinery dedicated to ferroptosis^24–26^ has been depicted in the Fig. 1 with the detailed description in the figure legend. In short, in the homeostatic conditions, enzymatically or non-enzymatically produced membrane lipid peroxides are effectively reduced to non-toxic alcohol derivatives, by the action of the Se-containing GPx4 enzyme^21^. The reducing power that drives the regeneration of GPx4 is GSH, a major non-enzymatic antioxidant in the cell^27^. Cellular concentration of GSH largely depends on the rate-limiting step in its biosynthesis catalysed by glutamate-cysteine ligase (GCL), or more precisely on the availability of the rate-limiting amino acid - CySH^28^. From its side, cysteine can be synthesized within the cell from methionine via transsulfuration pathway^29^. However, previous studies showed that this does not meet the requirements of highly proliferative and/or oxidatively compromised cells (such as cancer cells), which thus, largely rely on the import of this amino acid from the extracellular space^30^. Accordingly, the major transporter for the oxidized form (dominant form in the serum and almost exclusive form in the culture media) of CySH (cystine, CySSCy), known as Xc- system, seems to be consistently up-regulated within different types of cancer^31–40^. Xc- system, composed of a light transporter chain (xCT, *SLC7A11*) and a heavy chaperon subunit (CD98, *SLC3A2*), is an obligatory exchanger, allowing the import of CySSCy at the expense of glutamate export (1:1) (reviewed in ref. ^41^).

## Cell-born mechanisms of the resistance to ferroptosis induction

Until recently, the xCT-GSH-GPx4 axis has been seen as indispensable for prevention of ferroptosis. Hence, blocking CySSCy import via xCT (i.e. by erastin), synthesis of GSH (i.e. by buthionine sulfoximine, BSO) or GPx4 activity (i.e. by RAS-selective lethal 3, RSL3) proved to induce accumulation of membrane lipid peroxides within different cancer cell types, further inducing the loss of membrane assembly, structure, dynamics, and finally, cell death (ferroptosis)^42,43^. However, very recent studies point out that GSH-GPx4 part of the axis could be dispensable (reviewed in ref. ^44^), thanks to the presence of ubiquinol, as a reducing agent, and ferroptosis-supressor protein 1 (FSP1), as its regenerating enzyme, in the membrane compartments of the cell^45,46^. Even more, some additional roles (besides involvement in GSH biosynthesis) of CySH have been suggested as important for the process of ferroptosis, such as its incorporation into Coenzyme A—precursor of ubiquinol, via pantothenate pathway^44,47^, as well as its role in protein folding and endoplasmic reticulum homeostasis^48,49^. Lastly, an important and frequently overlooked feature of the GPx4 enzyme is its broad substrate specificity. Previous studies showed that GPx4 could efficiently use other thiol compounds (cysteine, among them) as a reducing power, instead of GSH, which can explain relative insensitivity of the cancer cells to GSH depletion^50–53^. In line with this is the study of Banjac and co-workers who demonstrated that lipid peroxidation can easily be prevented, even in the GSH-depleted conditions, thanks to the xCT-driven cystine/cysteine cycle^54^. In order to evaluate the contribution of these cell-born alternative pathways in the prevention of overwhelming accumulation of the lipid peroxides in the cell, additional studies are needed.

Although alternative cystine transporter (known as BAT1) exists^55,56^, to the best of our knowledge, its role in ferroptosis induction still has not been demonstrated, leaving the xCT alone in this context. The antiporter system Xc^−^ indeed seems to be indispensable transporter for CySSCy; however, studies suggest that the reduced form of this amino acid plays an important role in the resistance to xCT-dependent ferroptosis in vivo^34,57^. Although CySH in these conditions can be provided from the circulating blood, a growing number of studies suggest that the main source of cysteine and/or GSH to cancer cells are neighbouring cells. Interestingly, it is also suggested that cell-to-cell contact and its downstream signalling alone are able to induce ferroptosis resistance in the cancer cells, independently of cysteine-delivery. All of these aspects are discussed in more details below.

## Cell-to-cell contact

The importance of cell density for the sensitivity of mammalian cells to cysteine-starvation has been recognized as early as in the mid-20^th^ century, when Harry Eagle noticed that the cells become highly sensitive to cysteine removal, unless cell density reaches the “appropriate level”^58^. Later on, when the role of xCT was revealed, the same conclusion had been drawn once again in the comprehensive review article of Lewerenz and co-workers^41^. Interestingly, even in the case of GPx4 enzyme, it has been noted that GPx4-KO mouse embryonic fibroblasts are able to grow if seeded at higher density or in 3D spheroids, but not if seeded sparsely^22,59^.

Increased cell density is notably related to the resistance of solid tumours to many different chemotherapeutics, and in most cases, this has been linked to G1 cell cycle elongation time/arrest^60–64^. However, recent studies suggested that the density-related resistance might be a consequence of other molecular events, independently of the G1 cell cycle arrest^65–68^. This seems to be also the case for cysteine-starvation resistance, as the effects of increased density have been observed even before complete confluence (and arrested proliferation) is reached^34,69^. Also, Wenz and collaborator showed that density-related resistance to ferroptosis induced by tert-butyl hydroperoxide has not been a simple consequence of the growth arrest^69^.

### Hippo pathway involvement in the ferroptosis resistance: the role of YAP/TAZ

First papers dealing with the mechanistic insights of density-based resistance to ferroptosis appeared quite recently and suggested the Hippo pathway as a major player this event^70–74^. In brief, the main role of the highly conserved Hippo pathway is to restrict tissue growth in adults and to modulate cell proliferation, differentiation, and migration in developing organs, in response to different signals including cell-to-cell contact, cell polarity, energy status, mechanical clues, etc. The core of the Hippo signalling pathway consists of: the mammalian Ste20-like kinases 1/2 (MST1/2), large tumour suppressor 1/2 (LATS1/2), as well as two transcriptional co-activators: yes association protein (YAP) and its paralog WW domain containing transcription regulator 1 (TAZ). When activated, Hippo phosphorylation cascade starts by activation of MST1/2, which then, in cooperation with neurofibromin 2 (NF2 aka merlin), recruits and activates LATS1/2. From its side, LATS1/2 phosphorylates and inactivates YAP/TAZ, leading to their nuclear extrusion^75^. Moreover, it has been suggested that YAP/TAZ phosphorylation by LATS1/2 makes these transcriptional co-activators more prone to ubiquitination and degradation^76,77^.

The work of Wu and colleagues^70,71^ showed that the different sensitivity of a panel of human epithelial cancer cell lines to ferroptosis correlates with E-cadherin expression and Hippo pathway activity, seen through increased phosphorylation and decreased nuclear localization of YAP. The data also demonstrated that E-cadherin, NF2 or LATS1/2 knockdowns, have the same effect as low density, i.e. increase sensitivity to ferroptosis, while YAP(S127A), the mutant that cannot be phosphorylated by LATS1/LATS2, exhibited opposite effect even at a higher density or in spheroids. This further provides evidence for the functional link between E-cadherin, Hippo pathway and ferroptosis. Another study done on the highly ferroptosis-sensitive renal cell carcinoma (RCC) came to the same functional link, although focusing on the other Hippo pathway regulator—TAZ^73^.

**Fig. 2:**
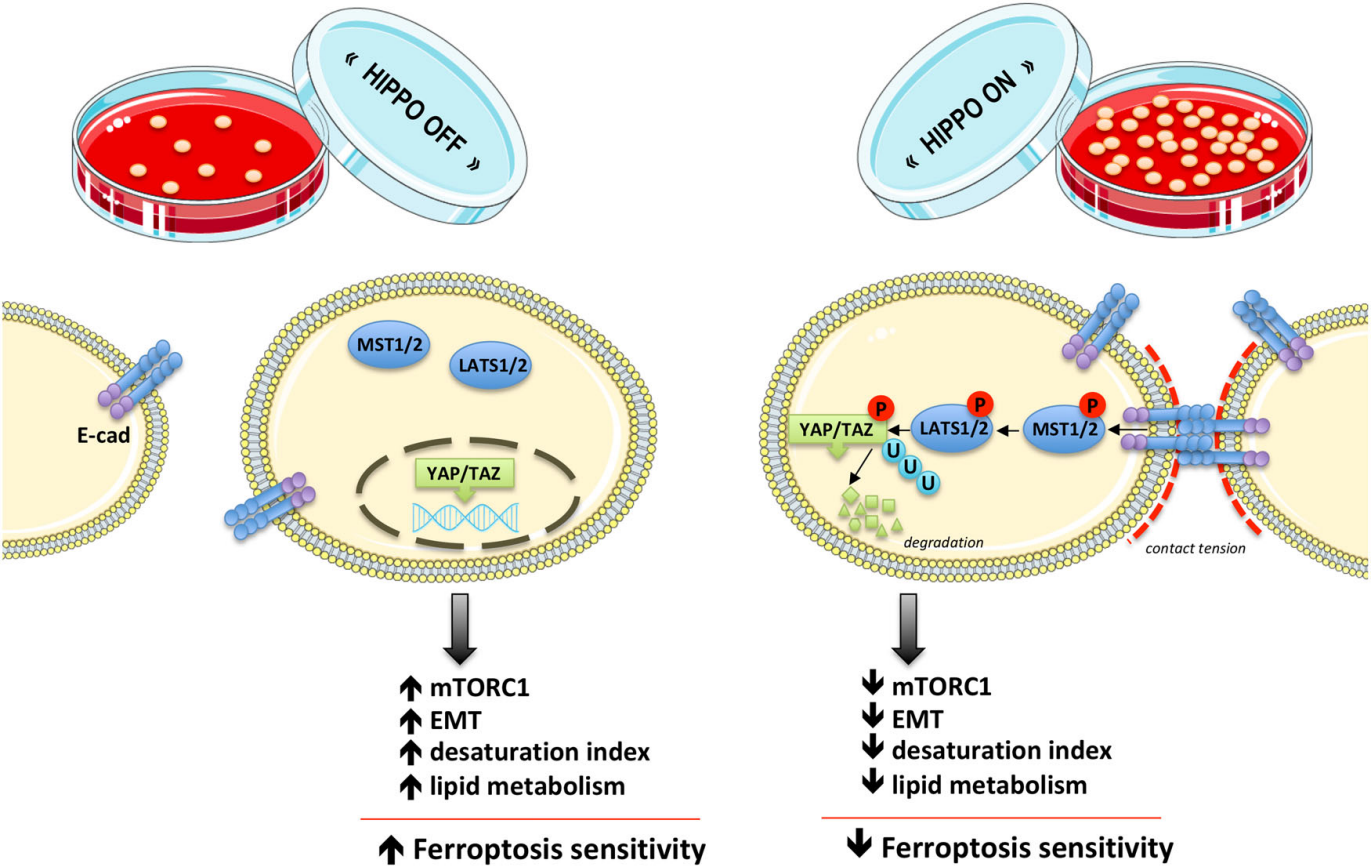
The effect of Hippo pathway on the sensitivity of cancer cells to ferroptosis. Cell-to-cell contact or other mechanical clues activate phosphorylation cascade of Hippo pathway that starts with mammalian Ste20-like kinases 1/2 (MST1/2), further activating large tumour suppressor 1/2 (LATS1/2) and resulting in phosphorylation, nuclear extrusion and degradation of two transcriptional co-activators: yes association protein (YAP) and its paralog WW domain containing transcription regulator 1 (TAZ). The effects of this molecular cascade branch to many intracellular signalling pathways (as described in the text) and ultimately result in decreased sensitivity of cancer cells to ferroptosis. This figure was created using Servier Medical Art templates, which are licensed under a Creative Commons Attribution 3.0 Unported License; https://smart.servier.com.

Although mutation in the Hippo components are uncommon in tumours, increasing number of data point out the importance of Hippo pathway (de)regulation in the cancer, which most likely stems out from tight interconnections between this and other crucial cellular pathways^75^. In the next four subsections, we will propose how these intricate signalling pathways might elicit resistance to ferroptosis (summarized in Fig. 2). However, it is very important to point out here and to keep in mind for the future research that the role of the Hippo pathway can be seemingly applied only to adherent cell, while its role for the hematopoietic cell (patho)physiology seems to be dispensable^78^. On the other side, the importance of the cell density for the growth and survival of lymphoma cells has been shown, just as their dependency on the proper thiol supply in the low-density conditions^79^. This clearly suggests that in the case of the cells in suspension, some other/additional mechanisms might be involved, and that the effects of the cell density on the cancer cell sensitivity to ferroptosis might go well beyond the Hippo pathway.

#### Hippo pathway and lipid peroxides

One of the major hallmarks of ferroptosis is the accumulation of membrane lipid peroxides. The main targets for this type of oxidative insult are polyunsaturated fatty acids (PUFAs) of membrane phospholipids containing bis-allylic hydrogen atoms that can be readily abstracted^80^. Hence, abundance of PUFAs largely influences the rate of peroxidation, and consequently, ferroptosis sensitivity. It has been shown that knockout of the genes involved in the biosynthesis and remodelling of PUFAs: long-chain-fatty-acid—CoA ligase 4 (ACSL4) or lysophosphatidylcholine acyltransferase 3 (LPCAT3) significantly decreases the sensitivity toward ferroptosis^81–84^. Interestingly, YAP/TAZ seem to be involved in the process of desaturation of the fatty acids. Specifically, stearoyl coenzyme A desaturase 1 (SCD1), an enzyme involved in the conversion of saturated into unsaturated fatty acids, mediates the release of YAP/TAZ from the β-catenin destruction complex. The data from lung adenocarcinoma samples revealed that SCD1 co-expresses with nuclear YAP/TAZ suggesting a clear correlation between expression and activity of these genes, and thus with the desaturation index of the cell^85–87^. Although this link between YAP/TAZ-dependent unsaturation index of the cell and ferroptosis sensitivity has still not been investigated, studies showing that E-cadherin-dependent YAP/TAZ nuclear extrusion decreases the accumulation of lipid peroxides^69–71,73^ suggest that this is something that deserves further and more in depth analysis.

Another way how Hippo components can affect the degree of lipid peroxidation is directly through influencing the ROS-producing systems. In the report of Yang and co-workers it has been shown that active and nuclear-localized TAZ regulates epithelial membrane protein 1 (EMP1) expression, which in turn increases NAD(P)H oxidase 4 (NOX4) expression in RCC. NOX4 is a ROS-producing enzyme whose involvement in lipid peroxidation has been noted in the previous studies^73,74^. An interesting observation of this study is that erastin did not affect GSH level in the TAZ knockout cells expressing low level of NOX4 (but did in WT cells with higher amount of NOX4), thus indicating that most of GSH reducing power in RCC has been used for amelioration of the NOX4-dependent oxidative damage^73^. Considering that NOX4 is an important source of ROS in the kidney^88,89^, the question that remains: is TAZ-EMP1-NOXs axis typical of renal redox homeostasis, and if so, are there other ROS-generating enzymes that might serve the same purpose in other tissues?

#### Hippo pathway and EMT

Long-lasting controversy about the importance of epithelial-to-mesenchymal transition (EMT) in cancer dissemination and metastasis in vivo, got its final proof relatively recently with epithelial lineage tracing in a mouse model^90^. On the other side, the role of EMT in cancer chemoresistance has been a widely accepted paradigm^91^. Due to high selective pressure, cells undergoing EMT have, from one side, low survival success rate, but from another, the ones that survive, do so by acquiring the resistance to many different environmental insults, including chemotherapy^91,92^. One of the rare studies challenging this dogma is the report of Viswanathan and colleagues, who showed that the therapy-resistant, highly mesenchymal cells are dependent on the xCT-GSH-GPx4 axis for their redox homeostasis, and are thus, extremely sensitive to ferroptosis^93^. Similar result has been obtained in our previous study, where sensitivity of epithelial pancreatic ductal adenocarcinoma (PDAC) cell line (Capan-2) to erastin increased upon induction of EMT with tumour growth factor β (TGF-β) treatment^34^. Although the higher sensitivity of mesenchymal phenotype to ferroptosis may look contra-intuitive at a first glance, when placed in the context of what has been aforementioned about cell-to-cell contact and resistance to ferroptosis, it makes much more sense.

One of the major characteristics of EMT is the loss of cell-to-cell contact and decreased expression of the canonical epithelial marker—E-cadherin^94^. Numerous studies dealing with the connections between Hippo pathway and EMT showed that overexpression of the Hippo pathway regulators, YAP/TAZ, is tightly related to the induction of the EMT^95–99^. Even more, some data points out the collaborative nature of the YAP/TAZ and EMT transcriptional factors^100^. Considering that PUFAs are essential for membrane fluidity, it is logical to assume that increased unsaturation index increases during EMT, when epithelial cells lose polarity and cell-cell adhesion and acquire mesenchymal morphology. Indeed, it has been observed that overexpression of enzymes such as ACSLs and SCD1 (involved in lipid metabolism and increased unsaturation index) induces EMT and increases migration/invasion of colorectal cancer cells^101^. Considering the already mentioned connection between Hippo pathway and unsaturation index, it seems plausible that this represent yet another way how the loss of cell-to-cell contact exposes cell vulnerability to lipid peroxidation, and consequently, ferroptosis. In this context, it might also be interesting to evaluate the possible interplay between hypoxia and the Hippo pathway, as hypoxia-inducible factor 1 (HIF-1) has been recognized as a key factor stimulating EMT and ferroptosis^102–105^.

According to all of this, it seems that the induction of EMT can deprive cells from protective mechanisms of cell-to-cell contact. However, the situation is not that simple. Indeed, Panzilius et al.^106^ recently observed that the overexpression of EMT transcription factor Twist1 or Snail1 was not sufficient to overcome density-related resistance in the human mammalian epithelial cells, although classical markers of mesenchymal phenotypes were detected. This might suggest that the signals induced by EMT transcription program alone are not sufficient to overcome density-induced resistance in all cases. However, it is worth noting that in this study the effects of EMT induction on GPx4 inhibition (ferroptosis) were not detected at any cell density, although the amount of this enzyme was 2.3–2.6 times higher in Twist1-expressing cells. Obviously, much more in depth analysis regarding the Hippo-(in)dependent EMT effects on ferroptosis sensitivity has to be undertaken in order to clarify whether, how and to what extent this pathway can influence ferroptotic process.

One very important aspect worth mentioning here and keeping in mind for the future research is that EMT, during cancer cell migration, does not necessary need to be complete. Namely, studies done by Jolly and collaborates^107^ showed that the migration and invasion of the cancer cells can be achieved through partial EMT. The clusters of cancer cells undergoing this non-compete EMT do partial de-differentiation while still maintaining cell-to-cell contacts (reviewed in refs. ^108,109^). Considering the topic of this review and all that had been said about sensitivity to ferroptosis in the context of cell-to-cell contacts, this study is of utmost importance for understanding relation between cell-to-cell contact, EMT and ferroptosis sensitivity.

#### Hippo pathway and mTORC1

Hippo and mechanistic target of rapamycin complex 1 (mTORC1) pathways are the two dominant regulators of normal organ growth and development. Although seemingly interconnected, the very crosstalk between them has not been fully illuminated. More precisely, different intersection points of this bidirectional regulation have been reported up to now. Thus, for both mTORC1 and mTORC2, it has been shown to positively regulate YAP stabilisation and signalling^110–112^, while different components of the Hippo pathway have been marked as regulating points of mTORC1. A recent report of Gan and co-workers^113^ showed that LATS1/2 suppresses mTORC1 activity by phosphorylation of S606 Raptor, and consequently preventing Raptor-Rheb interconnection. Similarly, NF2-deficient human meningioma cells and NF2-KD arachnoidal cells show rapamycin-sensitive constitutive mTORC1 activation^114^. On the other side, YAP/TAZ components of the Hippo pathway stimulate mTORC1 activity either through downregulation of PTEN (upstream negative regulator of mTORC1) or via upregulation of LAT1 (transporter of essential amino acid, necessary for mTORC1 activation) expression^115–117^.

Corroborating with this, our study showed, as mentioned previously, that highly epithelial PDAC cell line—Capan-2, has shown higher resistance to erastin-induced ferroptosis, both in term of kinetic and concentration, in comparison to mesenchymal MiaPaCa-2 PDAC cell line^34^. However, induction of EMT by TGF-β treatment equalized the sensitivity of these two PDAC cell lines. Interestingly, one of the sticking effects observed was the increase of mTORC1 activity upon TGF-β treatment, seen through increased phosphorylation status of mTORC1 pathway components. Here, we speculate that effects of TGF-β are the results of an enhanced protein synthesis and metabolism in general. Accordingly, protein synthesis inhibition by cycloheximide decreased sensitivity of MiaPaCa-2 cells to ferroptosis. Very similar results have been observed by Dixon and co-workers who, using live-cell time-lapse imaging, screened more than 1800 small molecules for ferroptosis and apoptosis inducers^118^. According to the data, ATP-competitive mTORC1, and mTORC1/PI3K inhibitors were associated with ferroptosis resistance; however is important to note that this was true only in the case of xCT-dependent ferroptosis, while no effects have been observed when GPx4 inhibitors were used for ferroptosis induction. Most likely, the explanation lies in the fact that mTORC1 inhibition decreases flux of amino acid into proteins and consequently redirects cysteine into GSH biosynthesis, which is confirmed by increased GSH content in the cells that were treated with mTORC1 or protein synthesis inhibitors^118^.

Although suppression of mTORC1 indeed seems like something that might be a problem in the cancer treatment context, it seems that the opposite is true for non-transformed cells. Namely, it has been shown that mouse cardiomyocytes isolated from cardiac-specific mTOR transgenic mice are less sensitive to Fe-donor, erastin, and RSL3-induced cell death in comparison with the control, while the opposite is observed in the case of cardiomyocytes isolated form cardiac-specific mTOR knockout mice^119^. This discrepancy in the effects of mTORC1 inhibition to ferroptosis sensitivity observed between transformed and non-transformed cells might be rooted in the well-known deregulation of mTORC1 activity upon neoplastic transformation. However, further studies are needed to clarify this issue.

#### Hippo pathway and proteotoxic stress

In the section where cell-born resistant mechanisms have been discussed, we briefly mentioned a very interesting study published recently by Harris and collaborators who showed that cancer cells display a wide range of sensitivity toward GSH depletion^49^. Although not directly stated in the paper, this suggests that GSH might be dispensable, and thus not the best target for ferroptosis induction in cancer. Using both pharmacological and genetic screening approaches the authors revealed the activity of some deubiquitinases as being central for the maintenance of protein homeostasis and thus survival upon GSH depletion. Similarly, Dixon et al.^48^ showed that ER stress and disturbed protein homeostasis are a very important part in xCT-dependent initiation of ferroptosis. In both cases, it has been suggested that cell death occurs as a consequence of deregulated antioxidant defence (GSH depletion), followed by proteotoxic stress (due to cysteine starvation-induced unfolded protein response/ER stress or deubiquitinase inhibition).

No connection in the literature have been made between this proteotoxicity-induced ferroptosis and cell-to-cell contact, but it is worth noting that the activity of Hippo pathway depends largely on ubiquitination–deubiquitination status (review in ref. ^120^). Namely, the activities of Hippo pathway components, including YAP/TAZ regulators, are under direct regulation of ubiquitination–deubiquitination process. Hence, it would be of great importance to illuminate how proteotoxic stress and disbalance in the ubiquitination–deubiquitination process may influence cell-to-cell physiology, and vice versa, especially in the case of epithelial cells resistant to xCT-dependent ferroptosis.

### Effect of cell-to-cell contact on the cancer metabolic rewiring

Metabolic rewiring due to the loss of cell-to-cell contact is mainly studied in the context of EMT and cancer metastasis^121^. Although the effects of these changes on ferroptosis sensitivity have not been extensively investigated, they might be of particular importance, especially as many already FDA-approved drugs targeting cancer metabolism could significantly improve the effectiveness of ferroptosis inducers. In the previously mentioned study of Panzilius et al.^106^, the authors showed that cell density significantly increases catabolism of lipids at the expense of increased vulnerability toward ferroptosis. More precisely, low density induces the release of free fatty acids from the lipid droplets, which further fuel ATP-production through mitochondrial β-oxidation. Hence, sensitivity to pharmacological/genetic invalidation of GPx4 in sparsely seeded human mammalian epithelial cells was reverted by inhibition of the adipose triglyceride lipase (ATGL)—the first enzyme in triacylglyceride hydrolysis, or by inhibition of β-oxidation. Similar effect has been observed in the case of mono-unsaturated fatty acid oleic acid treatment, which increases the content of cellular lipid droplet^122^. Taking into account what has been said about mTORC1 changes upon cell-to-cell contact lost, as well as its recognized, central role in lipid metabolism (for review see ref. ^123^), it is reasonable to assume that some of these metabolic changes stems from mTORC1 signalling, and consequently, could be manipulated upstream.

## Cell-to-cell interplay

Tumour microenvironment has long been recognized as an important factor in cancer progression and response to chemotherapy^124^. The specificities of this microenvironment significantly vary from one to another cancer type and generally depend on the types of surrounding non-transformed cells (fibroblasts, vascular endothelial cells, immune cells, etc.), as well as the characteristics of the extracellular matrix and milieu in which both transformed and non-transformed cells are deeply immersed (stiffness, oxygen, nutrient level, pH, etc.). The interaction between cancer and stromal cells is always bidirectional. Hence, although generally marked as “non-transformed”, stromal cells are significantly different to their counterparts in non-transformed tissues and organs. One of the characteristic examples are cancer-associate fibroblasts (CAFs), which seem to play an important role in cancer cell proliferation, invasion, and many other tumour-promoting activities (reviewed in ref. ^125^ and ref. ^126^), and their presence and activity in many different tumours correlates with patient poor prognosis^127^. Hence, it did not come as a surprise that stromal cells significantly influence response of cancer cells to ferroptosis induction as well.

**Fig. 3:**
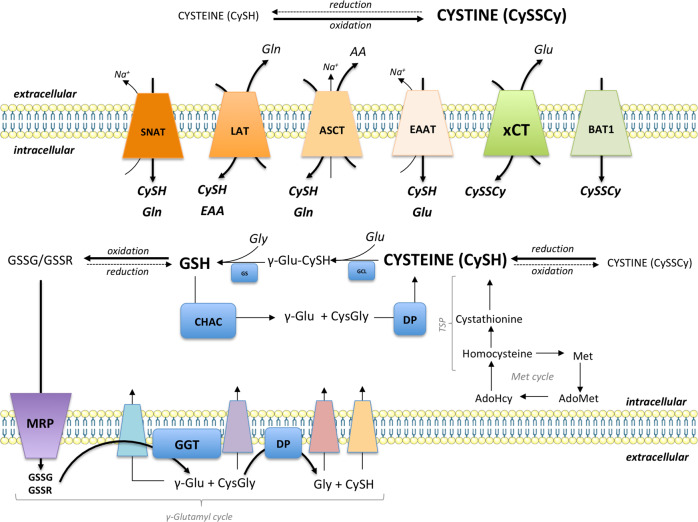
CySH/GSH cycle connecting intracellular and extracellular GSH and cysteine pools. Cysteine and cystine can be transported via Na^+^-dependent and Na^+^-independent transporters. Cystine imported by xCT and/or BAT1 is reduced in the intracellular milieu by GSH or thioredoxin. Alternatively, reduced form of cysteine can be directly imported or produced from homocysteine via the transsulfuration pathway. Cysteine is incorporated into GSH via the actions of γ-glutamylcysteine ligase (*GCL*) and glutathione synthetase (*GS*). Once oxidised, GSH is exported outside the cell via multidrug-resistance protein (MRP), where it is cleaved by the actions of GGT and a dipeptidase (DP). The cell can take each of the products of GSH cleavage, individually or as a dipeptide (γ-glutamyl cycle). Alternatively, GSH can be cleaved inside the cell by the action of CHAC and DP, that way serving as a cysteine intracellular pool. This figure was created using Servier Medical Art templates, which are licensed under a Creative Commons Attribution 3.0 Unported License; https://smart.servier.com.

**Fig. 4:**
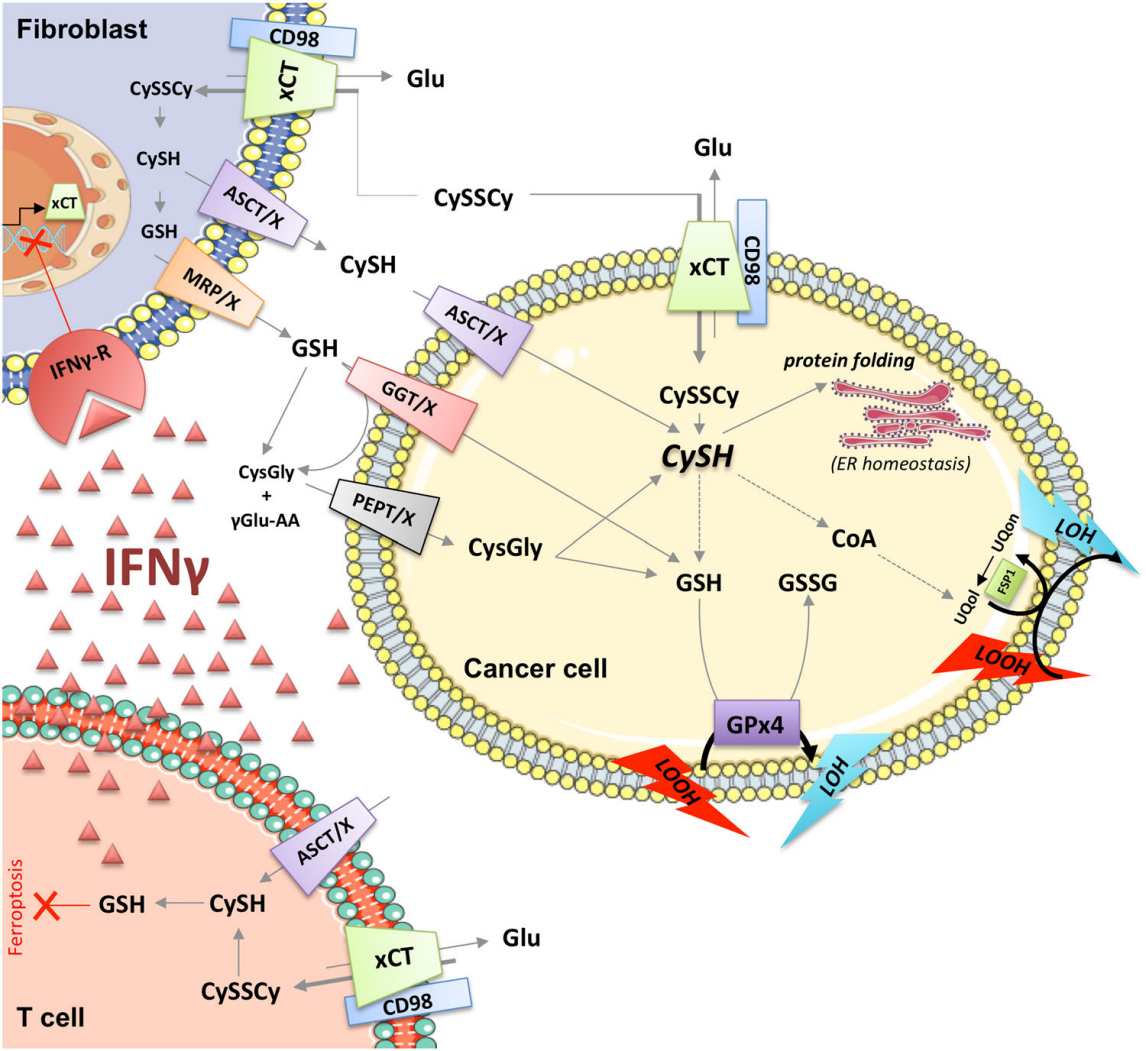
Cell-to-cell interplay (explained in the text). Glu, glutamate; CySH, cysteine; CySSCy, cystine; GSH, reduced glutathione; GSSG, oxidized glutathione; CoA, coenzyme A; UBol, ubiquinol; UBon, ubiquinone; FSP1, ferroptosis-suppression protein 1; LOOH, lipid peroxides; LOH, lipid alcohols; xCT, light chain of the system Xc- (cystine-glutamate transporter); CD98 chaperon heavy chain of the system Xc-; ER, endoplasmic reticulum; CysGly, cysteinyl-glycine; ASCT, alanine-serine-cysteine transporter; GGT, gamma-glutamyltranspeptidase; PEPT, peptide transporter; X, unknown transporter; γGlu-AA, gamma-glutamyl-amino acid; IFNγ, interferon gamma; IFNγR, IFNγ receptor. This figure was created using Servier Medical Art templates, which are licensed under a Creative Commons Attribution 3.0 Unported License; https://smart.servier.com.

Interesting data coming from the report of Wang and colleagues^128,129^ showed that resistance of breast cancer cells to platinum-based drugs comes from close interconnection between cancer cells and fibroblasts. Namely, cisplatin induces a severe drop in GSH level (most likely due to the mechanisms of cisplatin efflux from the cells that require its chelation with GSH), and thus, compromised survival of ovarian cancer cells^130^, which might explains the initial response of ovarian cancer to the therapy. However, this was easily prevented when cancer cell were co-cultured with fibroblasts. According to the data, fibroblasts were able to import oxidized (dominant) form of cysteine from the extracellular space, to reduce and make it available to cancer cells (Figs. 3, 4). This phenomenon might explain the in vivo resistance of highly (in vitro) sensitive xCT-KO cells shown by our group and others^34,57^. This CySH/GSH shuttle between xCT-expressing fibroblasts and cancer cells sounds rather logical, as knockdown of xCT in fibroblasts prevent fibroblast-mediated cisplatin resistance in ovarian cancer^129^. Even more, it seems that interferon-γ (IFN-γ) secreted from CD8^+^ T cells, has the same effect by inducing fibroblast-specific downregulation of xCT^129^. This is very similar concept to cystine/cysteine cycle observed in Burkitt’s lymphoma cells overexpressing xCT, which allow them to maintain the redox homeostasis even under GSH-depleting conditions^54^. However, Arensman and co-workers showed that resistance of xCT-KO cells is maintained even if the xenografts are implemented into xCT-KO mice^57^, raising the question whether xCT-mediated uptake of CySSCy and subsequent CySH/GSH shuttle is the only mechanism conferring ferroptosis resistance in the stromal compartment in vivo. A recent study of Zhang et al.^131^ suggests an alternative mechanism by which fibroblasts suppress ferroptosis and enhance chemoresistance in cancer cells: the secretion of miR-522 that leads to the suppression of arachidonate lipoxygenase 15 (ALOX15), an enzyme involved in lipid peroxidation. Altogether, the results suggest that the metabolic, but also cytokine, secretome of the stromal cells has to be taken in consideration when ferroptosis resistance is discussed.

Indeed, a key aspect of cell-to-cell interplay in stromal compartment is the interaction of immune and cancer cells. As mentioned previously, secretion of INF-γ by T cells has been suggested as ferroptosis stimulator in the case of ovarian cancer^128,129^. Interestingly, another way around has been shown as well. Nomi et al.^132^ observed that despite in vivo PDAC cells show resistance to xCT-induced ferroptosis, the genetic invalidation of xCT makes these cells more susceptible to immunotherapy. Namely, the clinical trials with antibodies against the two main checkpoint targets: programmed associate protein 1 (PD-1) and PD-1 ligand (PD-1L), did not show any benefit for pancreatic cancer patients, although PD-1L expression has been associated with poor prognosis in such patients^132^. The reason for this is still elusive, but a possible explanation may lay in the low immunogenic nature of PDAC cells due to the low mutation burden^133^. Interestingly, xCT-KO in pancreatic, but also colon cancer cell lines, enhanced the efficacy of checkpoint immunotherapy in a mouse xenograft model^57^, and it has been explained by a cysteine starvation-induced ER stress, which is necessarily connected with increased cell immunogenicity^48,134,135^.

## Conclusion

Almost a century-long fundamental research contributed to the emergence and contextualization of a new type of cell death—ferroptosis. At the very beginning of ferroptosis research, as we know it today, the mechanisms sounded very simple in comparison with the intricate signalling machinery employed for a defined apoptotic execution. However, almost ten years in, and we already can say that the situation with ferroptosis is not that simple as we might have thought. One of the most striking things that have been observed since the beginning, with now a better overall understanding of the underlying mechanisms, is the remarkable difference in the ferroptosis sensitivity between the cells seeded at different density. Understanding the physiology of cell-to-cell contact and how it provides resistance to oxidative insults, like lipid peroxidation, can tell us much more about the chemotherapeutical regimens that have to be used for maximal efficacy of ferroptosis inducers.
